# Data Resource Profile: National Child Oral Health Improvement Programmes for Chile

**DOI:** 10.1093/ije/dyac191

**Published:** 2022-10-20

**Authors:** Andrés Celis, David I Conway, Lorna M D Macpherson, Alex D McMahon

**Affiliations:** Community Oral Health, University of Glasgow Dental School, Glasgow, Scotland, UK; Faculty of Dentistry, University of Chile, Santiago, Chile; Community Oral Health, University of Glasgow Dental School, Glasgow, Scotland, UK; Community Oral Health, University of Glasgow Dental School, Glasgow, Scotland, UK; Community Oral Health, University of Glasgow Dental School, Glasgow, Scotland, UK

Key Features

- This article describes the process of establishing the ‘National Child Oral Health Improvement Programmes for Chile database’ via request, collation and linkage of national, regional and municipality level aggregated data (2008–19) of: child oral health programmes, interventions and activities; corresponding child dental caries outcomes; water fluoridation coverage; and area-based socioeconomic deprivation levels, to conduct a comprehensive evaluation of oral health complex interventions.
- The cohort consists of aggregated electronic dental health care routine data from all the 346 Chilean Municipalities from the year 2008 to 2019, including dental examinations of 4 117 537 children aged 0 to 6 years and primary care and child oral health programmes, activities and interventions linked to non-health data including population, community water fluoridation levels and coverage, and area-based deprivation indexes.
- Researchers who require access to the database linked to routine health information in conjunction with non-health information from the municipalities can download it from the ‘Enlighten: Research Data’ repository of the University of Glasgow: doi: 10.5525/gla.researchdata.1331.

## Data resource basics

Dental caries is one of the most prevalent diseases worldwide and represents a significant challenge for public health, especially in childhood.^1^^,^^2^ The Global Burden of Disease Study 2015 estimated that 7.8% of children suffer from untreated caries in deciduous teeth.^3^ Despite dental health improvements in the past decades, dental caries continues to be a global health problem particularlyly among disadvantaged groups, both in industrialized and in developing countries.^1^^,^^4^

Chile is facing up to the dental caries challenge in childhood by developing new public health programmes that aim to improve child oral health and reduce oral health inequalities. The last available cross-sectional national epidemiological study in 6-year-old children, published in 2007 and commissioned by the Chilean Ministry of Health, indicated that 70% of children had a history of dental caries. It also revealed marked inequalities by socioeconomic level and by geographical location,^5^ raising the need to improve oral health in childhood and reduce inequities in the distribution of the disease. The findings also highlighted the need for action towards a preventive approach, with upstream and downstream interventions and a strengthening of primary dental care, with actions developed jointly by health and educational teams and members of the community via a multidisciplinary approach. These new programmes are in addition to the national drinking water fluoridation programme that began in 1985 and that currently covers approximately 80% of the urban population of Chile, and to the dental activities performed in state-funded primary care clinics and hospitals.^6^^,^^7^ The new programmes have been developed by oral health policy makers, based on evidence from successful international examples, including the national child oral health improvement programme for Scotland—‘Childsmile’^8^^,^^9^—leading to the creation of the ‘Sembrando Sonrisas’ (Sowing Smiles) and the ‘Dental Diagnosis with Risk Approach’ programmes, and increasing the access to preventive and restorative dental activities performed in the primary care public clinics and hospitals of Chile.^10^

Briefly, the ‘Sembrando Sonrisas’ is a complex intervention with a preventive approach, offering specific activities focused on preschool-age children (from 2 to 5 years old) in state-funded nurseries and schools.^11^ The three main interventions of the programme are: (i) oral health examination by a dental team (dentist and dental nurse) in the community (classroom or other room of the educational establishment); (ii) training of educators and implementation of daily supervised toothbrushing in nurseries (including delivery of packages of fluoride toothpaste and toothbrushes for nurseries’ use); and (iii) applications of fluoride varnish in nurseries (FVA) (applied twice a year by dentists). After the pilot test, these interventions were collectively implemented nationally from 2015.^11^

The ‘Dental Diagnosis with Risk Approach’ programme had a national roll-out in 2017, targeted to children aged 0–9 years who attend the Chilean public health care system, with the objective of implementing a model to improve the efficiency and effectiveness of primary dental care for children at risk of oral diseases and contribute to preventing oral diseases in early ages.^12^ The programme is delivered in state-funded primary care community clinics and includes two main activities that are conducted within the same dental appointment: (i) dental examination and assessment of the risk of dental caries including medical history, dental examination, diet, oral hygiene, use of fluoride and family motivation/behaviours; and (ii) dentist-delivered prevention interventions and one-on-one education/advice to parents or caregivers in oral hygiene, and diet counselling. The recall frequency of these dental appointments depends on each child's caries risk.^12^

Using routine administrative health care service data is considered important in the evaluation of the reach and effectiveness of population-based public health interventions, including those aimed at addressing the challenge of dental caries at a population level.^13^ Such monitoring and evaluation can help inform modifications and future direction of public health programmes and policies.^14^^,^^15^

Despite having routine administrative dental health care and oral health improvement programmes data at the national level in Chile, to date there has not been any evaluation of child oral health programmes using these data. Although public institutions in Chile publish their information and make it available for research purposes,^16^ there has not been any linkage of oral health and dental service data with other sources of available commonly used non-health administrative data such as population, deprivation or fluoridated drinking water coverage.

This database includes a cohort of the 346 municipalities in the country with data from 4 117 537 unique dental examinations performed in children aged 0 to 6 years and from the activities carried out in the oral health programmes for children between the years 2008 and 2019. Each year there is one dental record per child, with no repeated measurements. These data were linked with non-health data aggregated at the national, regional and municipal levels.

## Data collected

### The National Child Oral Health Improvement Programmes for Chile database

The National Child Oral Health Improvement Programmes for Chile database was created through collating, extracting, processing and translating oral health electronic routine data and non-health datasets of all municipalities of the country. This was undertaken to enable evaluation of the new oral health policies implemented in Chile, improve their implementation, contribute to compliance with their objectives and serve as evidence for other countries. Primary care data from the Chilean Ministry of Health have been obtained from the Department of Health Statistics and Information of the Government of Chile, which collates routinely collected anonymized electronic health record data from each public health centre, whether hospital or community primary care clinic, which must compulsorily provide data on a monthly basis. All health activities and interventions performed on patients registered with the public health system (80% of the total population)^10^ are included in the dataset. According to the Chilean Data Governance Active Transparency Protocols,^16^ this information is publicly accessible and constitutes an open access database. Electronic health records databases for the years 2008 to 2019 were accessed and aggregated by year at national, regional and municipality levels in .mdb format from Microsoft Access Software (Microsoft Corporation; retrieved from https://office.microsoft.com/access). In this database, the progressive incorporation of electronic health records introduced missing data as, despite being mandatory since 2008, not all municipalities had the proper infrastructure and access to the network due to, among other reasons, geographical isolation and the technical feasibility of the internet. Table 1 shows the total number of dental examinations recorded in children from 0 to 6 years of age in the period included in the dataset.

**Table 1:** Total dental examinations records included in the dataset

Year	Municipalities with records	Dental examinations records						
		Age <1^a^	Age 1^a^	Age 2	Age 3^b^	Age 4	Age 5^b^	Age 6
2008	265	.	.	29 112	.	38 368	.	77 844
2009	263	.	.	35 664	.	44 728	.	92 166
2010	269	.	.	40 704	.	48 728	.	88 786
2011	274	.	.	48 090	.	56 697	.	95 958
2012	297	.	.	53 433	19 266	63 618	29 693	107 989
2013	309	.	.	59 364	27 214	70 756	35 149	111 913
2014	332	.	.	65 702	32 619	75 592	43 733	124 278
2015	331	.	.	77 110	44 204	85 934	52 333	140 045
2016	334	.	.	76 579	46 509	88 734	65 072	140 427
2017	340	26 475	32 277	87 706	55 136	86 249	66 950	141 753
2018	342	87 574	73 594	90 818	70 269	83 053	68 086	153 799
2019	343	98 047	73 516	73 596	59 991	68 578	60 126	125 833

a Electronic records of children aged <1 and 1 were introduced in 2017.

b Electronic records of children aged 3 and 5 were introduced in 2012.

Oral health activities and interventions data needed to be extracted, translated and indexed before being made available to the cohort. For this, a protocol was generated. The protocol consisted of the following.

- If the dataset of interest for the research corresponded to a subset of the original database, the variables of interest were selected, and the cases filtered according to the age range of interest for the cohort.
- The codebook of the files had to be accessed and the set of variables selected and then queried in Structured Query Language (SQL), with a standard code for all datasets from the same origin to select cases of interest,
- These were then exported in .xlsx format from Microsoft Excel Software (Microsoft Corporation; retrieved from https://office.microsoft.com/excel).
- Once the information was exported, the names of the variables were translated and coded from Spanish to English.
- Subsequently, the .xlsx file was imported into SAS 9.4 software (SAS Institute Inc., Cary, NC, USA.), where it was processed, indexed and saved as an .sas file. The database includes 186 variables and is constructed with a unit of analysis that combines the data's aggregation level and the time in calendar years. Since there are 346 municipalities in Chile, and to date 12 years of records are included in the database (from 2008 to 2019), 4152 municipality/years was the maximum number of analysis units.

### Data linkage

Municipality-level aggregated oral health electronic routine data were linked via Unique Municipal Territorial Codes^17^ to other existing data sources: (i) National Public Health Fund Insurance (FONASA) for the beneficiary population of the public health system datasets; (ii) Superintendence of Sanitary Services of the Ministry of Public Infrastructure of Chile for Fluoride Concentration and Coverage from the National Drinking Water datasets; (iii) National Institute of Statistics of Chile (INE) for Multidimensional Poverty Index, produced with the information collected from the Chilean National Socioeconomic Characterization Survey and population projections data from the National Census; and (iv) the Public Health Department of the University of Chile for Socioeconomic Development Index.

### Frequency of data collection

Routine oral health data collection is part of the clinical care of patients attending public health centres in Chile daily. Public health centres in Chile include state-funded primary care community clinics and hospital outpatient clinics, and they must submit data to the Department of Health Statistics and Information monthly. After checking the quality and anonymization of the data, the information must be uploaded to their website for public access on an annual basis. In the case of non-health data, this varies according to the regularity of the data collection. In the case of information on fluoridated drinking water, this is updated annually. Deprivation data are generally updated according to the regularity of the National Socioeconomic Characterization Survey, which is usually every 2 or 3 years.

### Measures

Table 2 gives an overview of the variables which are present the dataset. The database is structured based on municipality-level aggregation, organized by year of data registration. In addition, it separates the information into the following categories: (i) data collected from dental examinations carried out in public primary care clinics, relating to the diagnosis of the presence or absence of obvious decay; (ii) activities carried out in public primary care clinics;( iii) community water fluoridation data; (iv) the ‘Sembrando Sonrisas’ and ‘Dental Diagnosis with Risk Approach’ programmes; and (v) area-based information. In Chile, the World Health Organization guidelines for diagnosing dental caries are used as a reference.^18^ Historically, children's dmft scores are electronically recorded as categories, so based on this, the information was organized into children with and without obvious decay experience.

**Table 2:** Data elements of the National Child Oral Health Improvement Programmes for Chile Database

Linkage dataset	Coverage	Measurements
Child dental caries outcomes	2008 to 2019	Dental examinations
		Percentage of children with obvious caries experience
Activities performed in primary care public clinics	2008 to 2019	Preventive activities (fluoride varnish, sealants, toothbrushing education)
		Restorative activities (ionomer glass, composite, amalgams)
		Endodontics and exodontics
Community water fluoridation	2008 to 2019	Average annual fluoride concentration
		Fluoridated water coverage
Sembrando Sonrisas Programme	2015 to 2019	Sembrando Sonrisas reach and delivery
		Preventive interventions performed in nurseries and schools
Dental diagnosis with risk approach programme	2017 to 2019	Dental diagnosis with risk approach reach and delivery
		Dental diagnosis with risk approach survey and education
Area-based data	2008 to 2019	Region ID
		Unique municipal territorial code
		Socioeconomic development index
		Multidimensional poverty index
		Rurality index
		Total and public health system population

For information on community fluoridated water, each water company (in Chile, drinking water is managed by private companies) must periodically analyse the fluoride concentration in drinking water at each water treatment plant, issuing monthly and annual reports to the Superintendency of Sanitary Services of Chile which controls, verifies and manages these data.

## Data resource use

**Figure 1:**
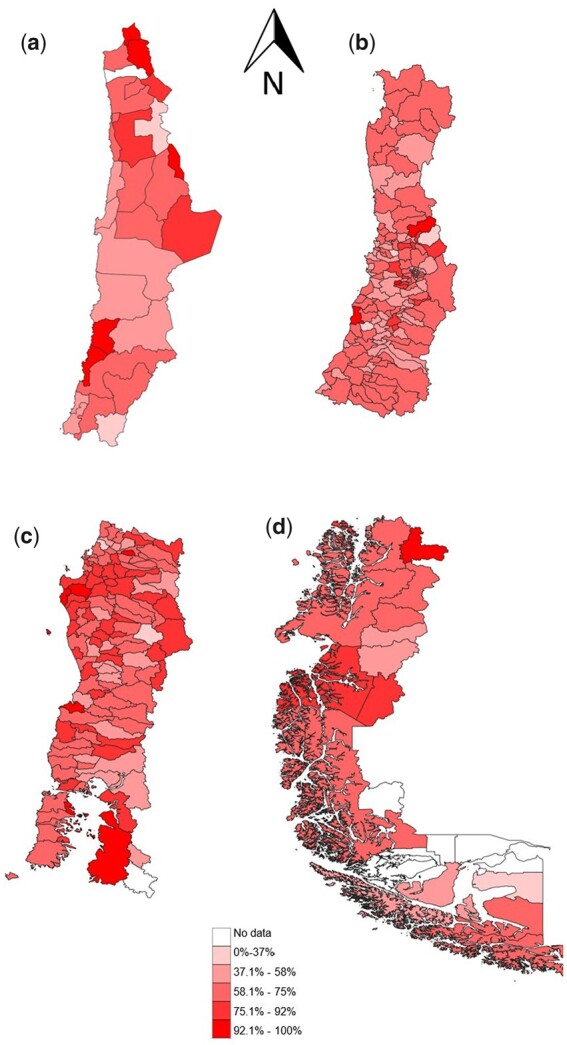
Caries experience of Chilean 6-year-olds by municipality, 2019. (a) North Macrozone, (b) North-centre Macrozone, (c) South-centre Macrozone, (d) South Macrozone

With the dental examinations data included in the database, it is possible to calculate outcomes so as to assess dental caries trends in Chilean children. Figure 1 presents the distribution of Chilean 6-year-olds with caries experience by municipality in the database for the year 2019. This information can also be used to investigate epidemiological associations between caries levels and the characteristics of municipalities, along with the impact of the activities and scope of oral health programmes for children, to provide evidence that can be used in decision making for public oral health policies in both the Chilean and international contexts.

## Strengths and weaknesses

### Strengths

The main strengths of this database are its size, breadth and scope, and that it contains more than a decade of information. The database to date includes aggregated data from more than 4 million children who were examined in primary public health centres between 2008 and 2019, along with the activities and dental interventions received from the National Child Oral Health Improvement Programs, including all municipalities from the beginning of the mandatory electronic registry. This is the first effort to link this routine health information with non-health records in Chile for the collaborative use of researchers.

The data quality is secured by the institutions that compile and make them available following current Chilean regulations. The government organizations in charge of controlling the information contained in this database have departments dedicated to data management and apply models of continuous improvement for information management, based on international standards, to contribute to decision making based on valid, comparable, reliable information that favours the integral wellbeing of the Chilean population and the development of public policies.^19^

### Weaknesses

In most databases there are missing data. This makes it difficult to describe the studied phenomena with precise statistics. As stated before, the progressive incorporation of electronic health records may introduce missing data. This phenomenon must be distinguished from the information not being captured, which could also account for some records being missing from the datasets. Of the total municipalities included (*n* = 346) and years analysed (12 years, from 2008 to 2019), there were a total of 4152 possible records of each variable included in which, to date, 81% of municipality/year have records for all variables in the database.

Another critical factor is that the information collected in health centres is not primarily intended for research, along with the fact that the dentists are not calibrated in their diagnoses and treatments. Hence, it is possible to assume an inherent variability due to the complexity of the aggregated records from routine health information.

## Data resource access

Researchers who require access to the database linked to routine health information in conjunction with non-health information from the municipalities can download it from the ‘Enlighten: Research Data’ repository of the University of Glasgow: doi: 10.5525/gla.researchdata.1331, or can directly contact the researcher in charge of the project [andres.celis@u.uchile.cl].

## Ethics approval

By the provisions of Chilean law on Data Protection and Private Life, the processing of secondary health data carried out by the Ministry of Health ensures the confidentiality of personal data to users who register as part of the Public Health System. In addition, patients' personal data are subject to procedures of dissociation of data, so that the information obtained is anonymized and cannot be associated with a specific or determinable person. The law of access to public information establishes that aggregated anonymized routine health data and the non-health information used in the assembly of this dataset can be publicly accessed under the principle of transparency of the Chilean Government public function. Therefore, no specific ethical approvals are needed for the use of these data.

## Data Availability

See Data Resource Access, above.
